# High-risk population's knowledge of risk factors and warning symptoms and their intention toward gastric cancer screening in Southeastern China

**DOI:** 10.3389/fpubh.2022.974923

**Published:** 2022-08-11

**Authors:** Zhiwen Huang, Wei Liu, Roy Rillera Marzo, Zhijian Hu, Li Ping Wong, Yulan Lin

**Affiliations:** ^1^Department of Epidemiology and Health Statistics, School of Public Health, Fujian Medical University, Fuzhou, China; ^2^Department of Community Medicine, International Medical School, Management and Science University, Shah Alam, Selangor, Malaysia; ^3^Global Public Health, Jeffrey Cheah School of Medicine and Health Sciences, Monash University Malaysia, Subang Jaya, Malaysia; ^4^Department of Social and Preventive Medicine, Faculty of Medicine, Centre for Epidemiology and Evidence-Based Practice, Universiti Malaya, Kuala Lumpur, Malaysia

**Keywords:** knowledge, attitude, intention, cancer screening, stomach cancer

## Abstract

**Background:**

As the incidence of gastric cancer (GC) increases sharply in adults aged over 40 years, screening of this high-risk population is important. This study aimed to explore knowledge level of GC related risk factors and symptoms, and to identify influencing factors associated with intention toward GC screening among people aged 40 years old and above in China.

**Methods:**

A cross-sectional, web-based survey was conducted among people aged 40 years old and above between October 2021 and March 2022 in Southeastern China. The participants' knowledge was assessed by a series of questions about risk factors (24-item scale) and warning symptoms (14-item scale).

**Results:**

A total of 2547 complete responses were received. The mean age was 47.72 (±7.20) years and near 60% were male. Respondents had a moderate level of knowledge about risk factors and warning symptoms of GC. The total mean knowledge score was 23.9 (±9.8) out of a possible score of 38. Majority (80%) of respondents reported intention to be screened for GC in the next 5 years. The most influential predictors of screening intention were income level (OR = 2.13, 95% CI: 1.36–3.32), perceived benefits (OR = 1.99, 95% CI: 1.33–2.73), perceived severity (OR = 1.68, 95% CI: 1.20–2.34), ever took GC screening (OR = 1.63, 95% CI: 1.28–2.08), perceived poor overall health (OR = 1.59, 95% CI: 1.19–2.11), and perceived barriers (OR = 1.56, 95% CI: 1.17–2.09). Other significant factors were ever diagnosed with chronic gastric diseases, total knowledge score, and cues-to-action. The major reasons for not willing to take screening were “endoscopy is uncomfortable” (29.6%), “worry about screening results” (23.6%), and “have no symptoms” (21.3%).

**Conclusion:**

High-risk population aged 40 years and above expressed high intention to receive GC screening. Intervention to improve health promotion and reduce the barriers to uptake of GC screening among high-risk populations in China is warranted.

## Introduction

Gastric cancer (GC) remains an important cancer worldwide and is responsible for over one million new cases in 2020 and an estimated 769,000 deaths (1). Eastern Asia and Central and Eastern Europe are regions with the highest incidence rate of GC in the world (2). In China, although the incidence and mortality have slightly decreased in the past two decades, high burden of GC still persists (3). The incidence and mortality rates of GC in China account for a staggering near 50% of the global burden (3, 4). GC is often asymptomatic in early stage, and the majority of patients were diagnosed with advanced stage, usually after they seek medical advice due to symptoms present (5). Likewise in China, more than 90% (6) of GC patients in clinics were presented at an advanced stage, in which the 5-year survival rate was only 35.1% (7). In contrast, the 5-year survival rate of patients with early GC after treatment exceeds 90% and can even be cured (8, 9).

Early detection of GC has great potential to improve survival and reduce disease mortality. Endoscopic screening for GC in moderate to high risk populations was found to be cost-effective (10), and it had been implemented in many countries with high incidence of GC (11, 12). Because the incidence of GC sharply increases after 40 years of age, regular screening is recommended for this target population in countries with high incidence of GC, such as Korea, Japan and China. The 5-year survival rate of GC is significantly lower in China than that of Japan and Korea (13), suggesting diagnosis delays among Chinese patients (14). Differences in screening rate coverage might partly explain the intercountry discrepancies of diagnosis delays. In Korea, National Cancer Screening Program (NCSP) has been initiated since 1999 to provide GC screening for patients 40 years or older every 2 years at no charge or 10% co-payment, depending on their insurance or income stratum (15). Upon implementation of GC screening program, screening rate in Korean has increased from 7.5% in 2002 to 47.3% in 2012 (16). As a result, more than 50% of GC in Korea were diagnosed at an early stage, compared to fewer than 10% in Western countries and China (17). Despite the serious burden of GC, there are no nationwide screening programs in China (14). Opportunistic screening with endoscopy in asymptomatic people is the primary practice in China (18). Compared with organized screening, opportunistic screening involves fewer formal decisions about whether to screen, whom to screen and at what intervals screening should be done (19). In 2005, China launched National Key Public Health Projects, and provided free endoscopic screenings for upper gastrointestinal cancer in more than 110 high-risk areas throughout the country. However, the estimated compliance rate (33.5%) was low (20). The national GC screening rate is still unknown in China. According to a recent cross-sectional study, the ever-screening rate of GC among adult Chinese was only 15.2% (21).

Similar to many countries worldwide, China has faced many obstacles in the introduction of GC screening, such as lack of knowledge related to GC and screening, high cost of screening, and negative attitude toward screening (21). GC is a multifactorial, multistep process (22). Host factors include blood group A, pernicious anemia, prior gastric surgery, family history, hereditary diffuse GC, and genetic syndromes. Smoking, salt, salty and smoked food, red meat, obesity, and low socioeconomic status are environmental factors. Moreover, infection with *Helicobacter pylori* and Epstein–Barr virus also play a role in gastric carcinogenesis (22, 23). Information on these risk factors helps characterize individuals at risk of GC during their lifetime and promote health-related behavior change. A recent survey from Korea (24) demonstrated that people with lower perceived risk of GC are less likely to take screening. This may primarily due to the fact that knowledge of the risk factors is a vital aspect in developing cancer risk perceptions and further influencing the participation in cancer screening (25, 26). In addition, knowledge about warning symptoms is critical for patients' timely medical care-seeking behavior. A recent study showed that knowledge about warning symptoms can lead to earlier presentation to medical care, which could result in earlier diagnosis and better outcomes (27). The presence of an abdominal lump, abdominal fullness and pain are typical warning symptoms of GC (28), which are easily mistaken as mild gastrointestinal disease. Economic problem was also suggested as a significant barrier. People in the lowest income level were less likely to undergo GC screening (21). Furthermore, negative attitudes toward GC screening, such as fear of screening procedure, fear of finding tumor, may also cause ignorance about screening (21, 29).

Fujian province, located in Southeastern part of China, is a well-known high-risk region of GC in China with higher incidence rate than the average national level (33.1/100,000 vs. 30.0/100,000) (30). Several cities in Fujian province have reported a 2-fold higher mortality rate than the national average level (49.47/100,000 vs. 21.9/100,000) (31). According to expert consensus in China, individuals aged at least 40 years from high-risk regions can be grouped as high-risk population of GC and regular screenings are recommended (6). To the best of our knowledge, no study on GC screening intention was carried out in high-risk population of China. Thus, the current study mainly aimed to investigate knowledge level of GC risk factors and symptoms as well as intention toward screening in Fujian province of China. Accurate information on factors associated with screening behaviors has important implications for health-related behavior change and may strengthen GC prevention and control.

## Methods

### Study design and participants

We commenced a cross-sectional, web-based anonymous survey using an online questionnaire during October 2021 and March 2022. Convenience sampling was conducted to recruit subjects for this study. The research team used WeChat (the most popular social media platform in China) to advertise and circulate the survey link to their network members. Network members were requested to distribute the survey invitation to all their contacts that satisfy the inclusion criteria. The inclusion criteria were that (1) aged 40 years and above; (2) living in Fuzhou, Putian, Quanzhou, Xiamen, and Zhangzhou city of Fujian province; (3) having no history of cancer. Upon completing the survey, each respondent providing a valid questionnaire was awarded an incentive of 5 Chinese Yuan (equivalent to 0.75 USD). In an attempt to reach a more comprehensive recipient coverage, we also encouraged participants to disseminate the survey link to all their contacts with a thank you note at the end. The participants were informed that their participation was voluntary, and consent was implied through their completion of the questionnaire. The reason for selecting these five cities was due to they are the major cities with the highest incidence of GC in Fujian Province. In total, the accumulated population of these five cities accounts for 73.43% of the total population in Fujian province (32).

### Instrument

The questionnaire was self-developed and pilot tested. Local experts of both epidemiologists and clinicians validated the content of the questionnaire. The survey consisted of four sections, which mainly assessed (1) demographic and general health; (2) knowledge about GC-related risk factors and warning symptoms; (3) history of treatment-seeking, and (4) attitudes and intention toward GC screening.

#### Demographic and general health

The first section of the questionnaire assessed participants' demographic characteristics such as age, gender, height, weight, highest education level, marital status, current residing location (urban/rural), current residing city, occupational types, and monthly average income. Participants were also asked if they ever knew any first-degree relatives, or any friends, neighbors, or colleagues who have been diagnosed with GC. For general health status, participants were asked if they “*Ever diagnosed with chronic gastric diseases (e.g., chronic gastritis, gastric ulcer, etc.)*”, perceived overall health, smoking, alcohol drinking, health insurance, and if they ever took GC screening.

#### Knowledge

The participants' knowledge was assessed by a series of questions about risk factors (24-item scale) and warning symptoms (14-item scale). The response options were “true,” “false,” or “don't know.” A correct response was given a score of one, and an incorrect or “don't know” response was scored zero. The total possible knowledge scores ranged from 0 to 38, with higher scores representing higher levels of knowledge. The median score was used to divide participants into high or low knowledge groups.

#### Attitudes

Health beliefs about GC screening was measured using the constructs from the Health Belief Model (HBM) (33). The questions probed perceived susceptibility to GC (three items), perceived severity of GC (three items), perceived benefits of GC screening (two items), perceived barriers to conduct GC screening (five items), and cues-to-action (three items). Perceived susceptibility queried participants about (1) general risk of a person having GC in their lifetime; (2) general risk of a person contracting *Helicobacter Pylori* in their lifetime, and (3) their own perceived risk of having GC. Perceived severity assessed participants' perception of harm of GC. Questions evaluating perceived benefits queried participants their views about the benefit of GC screening in early diagnosis and treatment of GC, and prognosis. Perceived barriers to conduct GC screening explored participants' concerns/hesitations when thinking of having screening. Cues-to-action questioned participants about motivation to conduct screening. The response options were “strongly agree,” “agree,” “disagree,” and “strongly disagree.”

A four-point scale was also used for questions about participants' intention to take GC screening in the next 5 years, namely “certainly yes,” “probably yes,” “probably no,” and “certainly no.” The domain reason for not being willing to take screening was also queried. Respondents were also requested to report their preferences of screening method by selecting one of the following options: “endoscopy,” “blood test,” “fecal examination,” and “none of them.”

### Sample size calculation

The minimal sample size was calculated based on the formula *N* = [$\mu_{\text{α}/2}^{2}$ × π × (1–π)]/δ^2^. The prevalence rate was 15% (π) based on the GC screening rate reported in the previous study (21), with a significant level set to be 0.05 (α), and allowable error as 0.03 (δ). The estimated minimal sample size was 544. In consideration of non-response rate, invalid questionnaire of 40%, a final target sample of 800 was determined.

### Statistical analyses

The reliability of the knowledge score was evaluated by assessing the internal consistency of the items representing the knowledge scores. Multivariable logistic regression was used to determine the factors influencing screening intention. All factors found to be statistically significant (*p*-value < 0.05) in the univariate regression analysis were entered into multivariable logistic regression analyses using a simultaneous forced-entry method. Odds ratio (OR), 95% confidence interval (95% CI) and *p*-values were calculated for each independent variable. The model fit of multivariable logistic regression analysis was assessed using the Hosmer-Lemeshow goodness-of-fit test (34). All *p-*values are based on a two-sided test with a statistical test level of α set at 0.05. All statistical analyses were performed using the Statistical Package for the Social Sciences (SPSS), version 26.0.

### Ethics and permission for data collection

Following the standards of Helsinki Declaration and its corresponding modifications or similar ethical principles, this study was carried out. The data was collected through an online survey where written informed consent was taken from each participant. Respondents who expressed their consent, after reading the aforementioned, to take part in the study by clicking either “Yes” or “No” were included in the study. Those who did not consent by clicking “No” were not included in the study. Ethics approval and permission for data collection were granted by the Medical Ethics Committee at the Fujian Medical University (FJMU No. 2020 [53]).

## Results

### Demographics characteristics of the participants

Between October 2021 and March 2022, a total of 2,547 completed responses were received. Supplementary Table 1 shows the demographics of our study participants compared with the adults aged 40 years and older population in Fujian. A summary of the characteristics of the participants is provided in the Table 1 and second column of **Table 3**. The mean age of study participants was 47.72 years (**±**7.20). A large proportion of participants were aged 40–50 years (78.2%). Near half of the participants lived in urban (56.9%) and had monthly average income > 5,000 RMB (750 USD) (48.0%). The highest education level is distributed nearly even in secondary school and below (33.3%), high school/technical school (35.1%), and university and above (31.6%). Only 18.8% of participants reported first-degree relatives had GC, while 40.6% were aware of their friends, neighbor, or colleagues had ever been diagnosed with GC. A total of 40.0% of participants reported a history of chronic gastric disease and 42.6% ever took GC screening.

**Table 1:** Demographic characteristics of respondents (*N* = 2,547).

**Characteristic**	**No**.	**%**
**Age, mean** **±SD**	47.72 ± 7.20	
**Age groups**
40–50	1,991	78.2
51–60	408	16.0
>60	148	5.8
**Body mass index (kg/m** ^**2**^ **)**		
<18.5	175	6.9
18.5–24.9	1,882	73.9
≥25.0	490	19.2
**Sex**		
Male	1,522	59.8
Female	1,025	40.2
**Educational level**		
Secondary school and below	849	33.3
High school/technical school	894	35.1
University and above	804	31.6
Monthly average income (RMB)^*^		
<2,000	291	11.4
2,000–5,000	1,034	40.6
>5,000	1,222	48.0
**Current residing location**		
Urban	1,448	56.9
Rural	1,099	43.1
**Current residing region**		
Fuzhou city	699	27.4
Putian city	425	16.7
Xiamen city	559	21.9
Zhangzhou city	430	16.9
Quanzhou city	434	17.1
**Occupation**
Professional and managerial	632	24.8
Office worker/service personnel	432	17.0
Industrial worker/Farmers/Others	770	30.2
Individual business/self-employed	443	17.4
Housewife/retired/unemployed	270	10.6

* 1 RMB = 0.15 USD.

### Knowledge about risk factor and warning symptoms of gastric cancer

**Figure 1:**
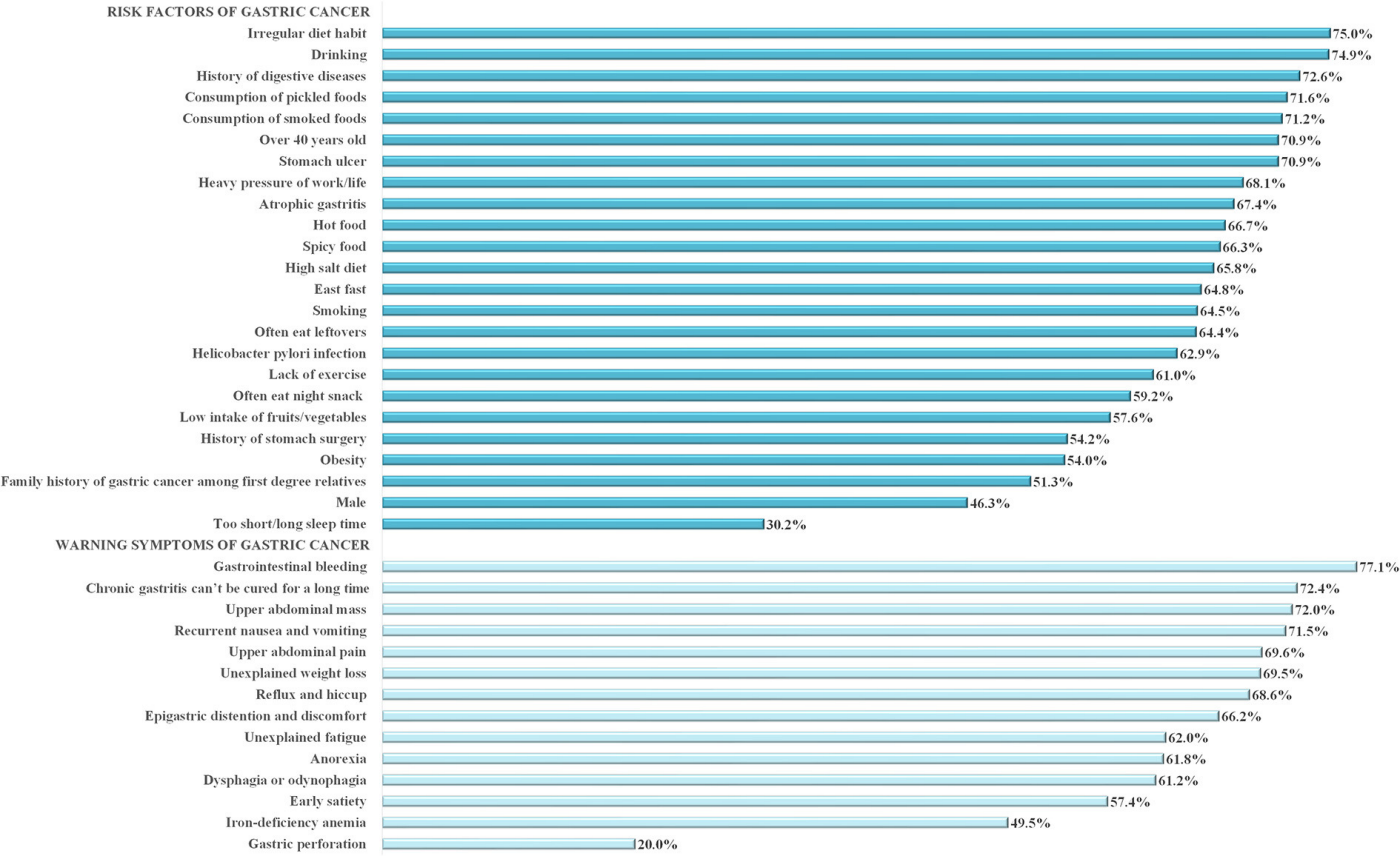
Percentages of correct responses to knowledge items (*N* = 2,547).

Figure 1 and Table 2 show the proportion of correct responses to all 38 knowledge items (24 items of risk factors and 14 items of warning symptoms). The 38 items for knowledge scores had a reliability (Cronbach's α) of 0.954. The mean and standard deviation (SD) for the total knowledge score was 23.9 (SD ± 9.8) out of a possible score of 38. The median was 25 (interquartile range, IQR, 17–33). Knowledge scores were categorized high or low based on median split; as such, a total of 1,209 (47.5%) were categorized as having a high score (25 to 38) and 1,338 (52.5%) had a low score (0–24).

**Table 2:** Respondents' knowledge about risk factors and warning symptoms of gastric cancer.

**Category**	**Yes, *n* (%)**	**No, *n* (%)**	**Don't know, *n* (%)**
**Risk factors of gastric cancer**			
Aged 40 years and over	1,806 (70.9)	390 (15.3)	351 (13.8)
Male	1,178 (46.3)	848 (33.3)	521 (20.5)
*Helicobacter pylori* infection	1,603 (62.9)	467 (18.3)	477 (18.7)
Stomach ulcer	1,806 (70.9)	463 (18.2)	278 (10.9)
Atrophic gastritis	1,716 (67.4)	505 (19.8)	326 (12.8)
Family history of gastric cancer among first degree relatives	1,306 (51.3)	902 (35.4)	339 (13.3)
High salt diet	1,677 (65.8)	582 (22.9)	288 (11.3)
Consumption of pickled foods	1,824 (71.6)	534 (21.0)	189 (7.4)
Consumption of smoked foods	1,813 (71.2)	550 (21.6)	184 (7.2)
Irregular diet habit	1,910 (75.0)	488 (19.2)	149 (5.9)
Often eat leftovers	1,639 (64.4)	668 (26.2)	240 (9.4)
Smoking	1,644 (64.5)	634 (24.9)	269 (10.6)
Alcohol drinking	1,907 (74.9)	464 (18.2)	176 (6.9)
High pressure of work/life	1,734 (68.1)	547 (21.5)	266 (10.4)
Often eat night snack	1,507 (59.2)	738 (29.0)	302 (11.9)
Lack of exercise	1,553 (61.0)	669 (26.3)	325 (12.8)
Low intake of fruits/vegetables	1,468 (57.6)	806 (31.6)	273 (10.7)
Too short/long sleep time	1,401 (55.0)	768 (30.2)	378 (14.8)
Obesity	1,375 (54.0)	801 (31.4)	371 (14.6)
History of digestive diseases	1,848 (72.6)	522 (20.5)	177 (6.9)
History of stomach surgery	1,381 (54.2)	899 (35.3)	267 (10.5)
Consumption of spicy food	1,689 (66.3)	636 (25.0)	222 (8.7)
Consumption of hot food	1,700 (66.7)	634 (24.9)	213 (8.4)
East fast	1,650 (64.8)	616 (24.2)	281 (11.0)
**Warning symptoms of gastric cancer**		
Gastrointestinal bleeding	1,965 (77.1)	398 (15.6)	184 (7.2)
Recurrent nausea and vomiting	1,821 (71.5)	469 (18.4)	257 (10.1)
Unexplained weight loss	1,770 (69.5)	514 (20.2)	263 (10.3)
Unexplained fatigue	1,579 (62.0)	603 (23.7)	365 (14.3)
Epigastric distention and discomfort	1,685 (66.2)	527 (20.7)	335 (13.2)
Upper abdominal mass	1,834 (72.0)	430 (16.9)	283 (11.1)
Upper abdominal pain	1,772 (69.6)	475 (18.6)	300 (11.8)
Anorexia	1,573 (61.8)	626 (24.6)	348 (13.7)
Dysphagia or odynophagia	1,559 (61.2)	679 (26.7)	309 (12.1)
Early satiety	1,461 (57.4)	702 (27.6)	384 (15.1)
Reflux and hiccup	1,746 (68.6)	512 (20.1)	289 (11.3)
Chronic gastritis can't be cured for a long time	1,845 (72.4)	459 (18.0)	243 (9.5)
Iron-deficiency anemia	1,260 (49.5)	798 (31.3)	489 (19.2)
Gastric perforation	1,788 (70.2)	509 (20.0)	250 (9.8)

The most highly recognized risk factors were “irregular diet habit” (75.0%), and “alcohol drinking” (74.9%), followed by “history of digestive disease” (72.6%), “consumption of pickled food” (71.6%), “consumption of smoked food” (71.2%), “aged 40 years and above” (70.9%), and “stomach ulcer” (70.9%). The least recognized risk factor was “male” (46.3%). In particular, only 56.7% of male respondents (data not shown) were aware of this inherent risk. Meanwhile, majority of participants wrongly regarded “too short/long sleeping time” (55.0%) as a risk factor of GC. The most highly recognized warning symptoms were “gastrointestinal bleeding” (77.1%), followed by “chronic gastritis can't be cured for a long time” (72.4%), “upper abdominal pain” (72.0%) and “recurrent nausea and vomiting” (71.5%). The least recognized warning symptoms were “early satiety” (57.4%) and “hypoferric anemia” (49.5%), while 70.2% of respondents wrongly considered “gastric perforation” (70.2%) as a warning symptom.

### Gastric cancer screening intention and its influencing factors

**Figure 2:**
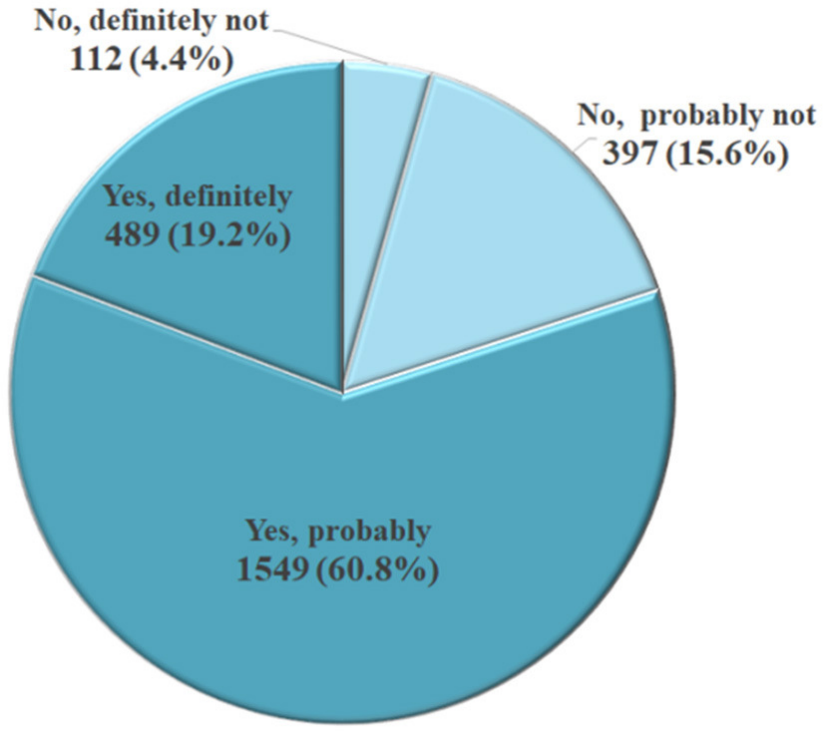
Intention to take gastric cancer screening in the next 5 years (*N* = 2,547).

Figure 2 shows the proportions of intention to take screening in the next 5 years. In total, 80.0% (*n* = 2,038) of participants reported “certainly yes/probably yes” and 20.0% (*n* = 509) reported “certainly no/probably no” regarding their intention to screen in the next 5 years (Figure 2).

Results of univariate and multivariable logistic regression were presented in Table 3. Multivariable logistic regression showed that monthly income > 5,000 RMB (OR = 2.13, 95% CI: 1.36–3.32) was the most robust factor associated with screening intention. Respondents that perceived their own overall health as “fair/poor/very poor” (OR = 1.59, 95% CI: 1.19–2.11), ever took GC screening (OR = 1.63, 95% CI: 1.28–2.08) had more than 50% higher odds of intention to conduct screening. The odds of intention to conduct screening were also higher among respondents who were ever diagnosed with chronic gastric diseases (OR = 1.30, 95% CI: 1.01–1.68), and those had high score of total knowledge (OR = 1.46, 95% CI: 1.16–1.84). Results of HBM indicate that the following five components were significantly associated with screening intention, including perceived susceptibility (risk of getting GC is high, OR = 1.44, 95% CI: 1.11–1.85), perceived severity (afraid of getting GC, OR = 1.68, 95% CI: 1.20–2.34), perceived benefit (GC screening is effective in saving life, OR = 1.99, 95% CI: 1.33–2.73), perceived barriers (endoscopy is uncomfortable, OR = 1.56, 95% CI: 1.17–2.09), and cues-to-action (only take screening when it is free of charge, OR = 1.47; 95% CI: 1.08–2.00).

**Table 3:** Factors associated with intention to take gastric cancer screening in the next 5 years (*N* = 2,547).

	**Frequency (%)**	**Univariate analysis**				Multivariable logistic regression^*^	
		**Intention**			***p*-value**	**Yes vs. No**	***p*-value**
		**Yes** ***n* = 2,038**	**No *n* = 509**	**Unadjusted** **OR** **(95% CI)**		**OR (95% CI)**	
**Baseline demographic**							
**Age group (years old)**							
40–50	1,991 (78.2)	1,616 (81.2)	375 (18.8)	2.01(1.39–2.89)	0.001	1.36 (0.84–2.21)	0.206
51–60	408 (16.0)	321 (78.7)	87 (21.3)	1.72 (1.13–2.61)		1.24 (0.75–2.05)	0.404
>60	148 (5.8)	101 (68.2)	47 (31.8)	Reference		Reference	
**Sex**							
Male	1,522 (59.8)	1,209 (79.4)	313 (20.6)	0.91 (0.75–1.12)	0.372		
Female	1,025 (40.2)	829 (80.9)	196 (19.1)	Reference			
**Body mass index (kg/m** ^**2**^ **)**							
<18.5	175 (6.9)	135 (77.1)	40 (22.9)	0.88 (0.58–1.33)	0.538		
18.5–24.9	1,882 (73.9)	1,514 (80.4)	368 (19.6)	1.07 (0.84–1.37)			
≥25.0	490 (19.2)	389 (79.4)	101 (20.6)	Reference			
**Highest education level**							
Primary school and below	283 (11.1)	197 (69.6)	86 (30.4)	Reference	*p* < 0.001	Reference	
Secondary school	566 (22.2)	450 (79.5)	116 (20.5)	1.69 (1.22–2.35)		0.93 (0.60–1.46)	0.762
High school/technical school	894 (35.1)	729 (81.5)	165 (18.5)	1.93 (1.42–2.62)		1.33 (0.92–1.91)	0.129
University and above	804 (31.6)	662 (82.3)	142 (17.7)	2.04 (1.49–2.78)		1.05 (0.78–1.42)	0.759
**Marital status**							
Married	2,240 (87.9)	1,821 (81.3)	419 (18.7)	1.80 (1.38–2.36)	*p* < 0.001	1.30 (0.92–1.83)	0.136
Unmarried/divorced/separated/widowed	307 (12.1)	217 (70.7)	90 (29.3)	Reference		Reference	
**Current residing location**							
Urban	1,448 (56.9)	1,180 (81.5)	268 (18.5)	1.24 (1.02–1.50)	0.033	0.86 (0.67–1.09)	0.212
Rural	1,099 (43.1)	858 (78.1)	241 (21.9)	Reference		Reference	
**Current residing region**							
Fuzhou city	699 (27.4)	562 (80.4)	137 (19.6)	0.97 (0.72–1.31)	0.004	0.79 (0.56–1.11)	0.116
Putian city	425 (16.7)	344 (80.9)	81 (19.1)	1.00 (0.72–1.41)		1.41 (0.94–2.13)	0.098
Xiamen city	559 (21.9)	465 (83.2)	94 (16.8)	1.17 (0.84–1.62)		0.91 (0.64–1.31)	0.621
Zhangzhou city	430 (16.9)	316 (73.5)	114 (26.5)	0.66 (0.48–0.90)		0.79 (0.55–1.12)	0.186
Quanzhou city	434 (17.0)	351 (80.9)	83 (19.1)	Reference		Reference	
**Occupation**							
Professional and managerial	632 (24.8)	519 (82.1)	113 (17.9)	1.83 (1.31–2.59)	*p* < 0.001	0.86 (0.53–1.40)	0.554
Office worker/Service personnel	432 (17.0)	371 (85.9)	61 (14.1)	2.43 (1.67–3.54)		1.22 (0.75–2.01)	0.425
Industrial worker /Farmers/Others	770 (30.2)	592 (76.9)	178 (23.1)	1.33 (0.97–1.81)		0.89 (0.59–1.35)	0.580
Individual business/ Self-employed	443(17.4)	363 (81.9)	80 (18.1)	1.81 (1.27–2.59)		0.98 (0.61–1.58)	0.930
Housewife/Retired/Unemployed	270(10.6)	193 (71.5)	77 (28.5)	Reference		Reference	
**Monthly average income (RMB)**							
<2,000	291 (11.4)	189 (64.9)	102 (35.1)	Reference	*p* < 0.001	Reference	
2,000–5,000	1,034 (40.6)	827 (80.0)	207 (20.0)	2.16 (1.62–2.87)		1.70 (1.15–2.50)	0.008
>5,000	1,222 (48.0)	1,022 (83.6)	200 (16.4)	2.76(2.08–3.67)		2.13 (1.36–3.32)	0.001
**Experience with gastric cancer**							
**Ever known any first-degree relatives has had gastric cancer**							
Yes	479 (18.8)	406 (84.8)	73 (15.2)	1.49 (1.13–1.95)	0.004	1.05 (0.76–1.45)	0.772
No	2,068 (81.2)	1,632 (78.9)	436 (21.1)	Reference		Reference	
**Ever known any friends, neighbor, colleagues have had gastric cancer**							
Yes	1,033 (40.6)	878 (85.0)	155 (15.0)	1.73 (1.40–2.13)	*p* < 0.001	1.09 (0.85–1.40)	0.483
No	1,514 (59.4)	1,160 (76.6)	354 (23.4)	Reference		Reference	
**Health characteristics**							
**Ever diagnosed with chronic gastric diseases (e.g., chronic gastritis, gastric ulcer, etc.)**							
Yes	1,020 (40.0)	872 (85.5)	148 (14.5)	1.82 (1.48–2.25)	*p* < 0.001	1.30 (1.01–1.68)	0.041
No	1,527 (60.0)	1,166 (76.4)	361 (23.6)	Reference		Reference	
**Perceived overall health**							
Very good	429 (16.8)	317 (73.9)	112 (26.1)	Reference	*p* < 0.001	Reference	
Good	697 (27.4)	546 (78.3)	151 (21.7)	1.28 (0.97–1.69)		1.10 (0.81–1.50)	0.542
Fair/poor/very poor	1,421 (55.8)	1,175 (82.7)	246 (17.3)	1.69 (1.31–2.18)		1.59 (1.19–2.11)	0.002
**Smoking**							
Yes	829 (32.5)	682 (82.3)	147 (17.7)	1.24 (1.00–1.53)	0.049	1.05 (0.82–1.34)	0.696
No	1,718 (67.5)	1,356 (78.9)	362 (21.1)	Reference		Reference	
**Alcohol drinking**							
Yes	627 (24.6)	505 (80.5)	122 (19.5)	1.05 (0.83–1.31)	0.704		
No	1,920 (75.4)	1,533 (79.8)	387 (20.2)	Reference			
**Health insurance**							
Yes	2,276 (89.4)	1,850 (81.3)	426 (18.7)	1.92 (1.45–2.53)	*p* < 0.001	1.16 (0.82–1.34)	0.403
No	271 (10.6)	188 (69.4)	83 (30.6)	Reference		Reference	
**Ever took gastric cancer screening**							
Yes	1,086 (42.6)	943 (86.8)	143 (13.2)	2.20 (1.78–2.73)	*p* < 0.001	1.63 (1.28–2.08)	*p* < 0.001
No	1,461 (57.4)	1,095 (74.9)	366 (25.1)	Reference		Reference	
**Knowledge of risk factors and warning symptoms**							
Total knowledge score							
Low score (0–24)	1,209 (47.5)	889 (73.5)	320 (26.5)	Reference	*p* < 0.001	Reference	
High score (25–38)	1,338 (52.5)	1,149 (85.9)	189 (14.1)	2.19 (1.79–2.67)		1.46 (1.16–1.84)	0.001
**Health beliefs**							
**Perceived susceptibility**							
In general, a person has a high risk of having gastric cancer in their lifetime					*p* < 0.001		
Strongly agree/agree	1,410 (55.4)	1,199 (85.0)	211 (15.0)	2.02 (1.66–2.46)		1.44 (1.11–1.85)	0.005
Disagree/strongly disagree	1,137 (44.6)	839 (73.8)	298 (26.2)	Reference		Reference	
I may have gastric cancer							
Strongly agree/agree	967 (38.0)	833 (86.1)	134 (13.9)	1.94 (1.60–2.40)	*p* < 0.001	1.17 (0.90–1.52)	0.247
Disagree/strongly disagree	1,580 (62.0)	1,205 (76.3)	375 (23.7)	Reference		Reference	
In general, a person has a high risk of infecting *Helicobacter pylori* infection in their lifetime					
Strongly agree/agree	1,676 (65.8)	1,404 (83.8)	272 (16.2)	1.93 (1.58–2.35)	*p* < 0.001	0.91 (0.70–1.19)	0.491
Disagree/strongly disagree	871 (34.2)	634 (72.8)	237 (27.2)	Reference		Reference	
**Perceived severity**							
Harms of gastric cancer are severe							
Strongly agree/agree	2,273 (89.2)	1,870 (82.3)	403 (17.7)	2.93 (2.24–3.82)	*p* < 0.001	0.98 (0.64–1.48)	0.906
Disagree/strongly disagree	274 (10.8)	168 (61.3)	106 (38.7)	Reference		Reference	
Mortality rate of gastric cancer is very high					
Strongly agree/agree	1,995 (78.3)	1,651 (82.8)	344 (17.2)	2.05 (1.65–2.54)	*p* < 0.001	1.16 (0.87–1.53)	0.309
Disagree/strongly disagree	552 (21.7)	387 (70.1)	165 (29.9)	Reference		Reference	
I am afraid of getting gastric cancer							
Strongly agree/agree	2,172 (85.3)	1,802 (83.0)	370 (17.0)	2.87 (2.26–3.64)	*p* < 0.001	1.68 (1.20–2.34)	0.002
Disagree/strongly disagree	375 (14.7)	236 (62.9)	139 (37.1)	Reference		Reference	
**Perceived benefit**							
Screening is highly effective in early diagnosis and early treatment of gastric cancer						
Strongly agree/agree	2,288 (89.8)	1,884 (82.3)	404 (17.7)	3.18 (2.43–4.17)	*p* < 0.001	1.10 (0.71–1.70)	0.673
Disagree/strongly disagree	259 (10.2)	154 (59.5)	105 (40.5)	Reference		Reference	
Gastric cancer screening highly effective in reducing death rate							
Strongly agree/agree	2,229 (87.5)	1,853 (83.1)	376 (16.9)	3.54 (2.76–4.54)	*p* < 0.001	1.99 (1.33–2.73)	*p* < 0.001
Disagree/strongly disagree	318 (12.5)	185 (58.2)	133 (41.8)	Reference		Reference	
**Perceived barriers**							
I'm afraid screening will find something bad				
Strongly agree/agree	1,772 (69.6)	1,435 (81.0)	337 (19.0)	1.22 (0.99–1.49)	0.065		
Disagree/strongly disagree	775 (30.4)	603 (77.8)	172 (22.2)	Reference			
Screening is only necessary when symptoms present					
Strongly agree/agree	1,442 (56.6)	1,102 (76.4)	340 (23.6)	Reference	*p* < 0.001	Reference	
Disagree/strongly disagree	1,105 (43.4)	936 (84.7)	169 (15.3)	1.71 (1.39–2.10)		1.29 (1.00–1.65)	0.046
Endoscopy is uncomfortable				
Strongly agree/agree	1,879 (73.8)	1,530 (81.4)	349 (18.6)	Reference	0.003	Reference	
Disagree/strongly disagree	668 (26.2)	508 (76.0)	160 (24.0)	1.38 (1.12–1.71)		1.56 (1.17–2.09)	0.002
Cost of endoscopy is very high							
Strongly agree/agree	1,588 (62.3)	1,252 (78.8)	336 (21.2)	0.82 (0.67–1.01)	0.057		
Disagree/strongly disagree	959 (37.7)	786 (82.0)	173 (18.0)	Reference			
It is difficult and time-consuming to have an appointment for endoscopy screening.							
Strongly agree/agree	1,692 (66.4)	1,337 (79.0)	355 (21.0)	0.83 (0.67–1.02)	0.077		
Disagree/strongly disagree	855 (33.6)	701 (82.0)	154 (18.0)	Reference			
**Cues-to-action**							
I only take screening when it's free							
Strongly agree/agree	1,170 (45.9)	869(74.3)	301 (25.7)	Reference	*p* < 0.001	Reference	
Disagree/strongly disagree	1,377 (54.1)	1,169(84.9)	208 (15.1)	1.95 (1.60–2.37)		1.47 (1.08–2.00)	0.013
I only take screening when it can be covered by medical insurance							
Strongly agree/agree	1,328 (52.1)	1,003 (75.5)	325 (24.5)	Reference	*p* < 0.001	Reference	
Disagree/strongly disagree	1,219 (47.9)	1,035 (84.9)	184 (15.1)	1.82 (1.49–2.23)		1.24 (0.90–1.70)	0.182
I only take screening when doctor recommends							
Strongly agree/agree	1,804 (70.8)	1,423 (78.9)	381 (21.1)	Reference	0.026	Reference	
Disagree/strongly disagree	743 (29.2)	615 (82.8)	128 (17.2)	1.29 (1.03–1.61)		0.91 (0.67–1.23)	0.530

* Hosmer & Lemeshow test, chi-square: 303.947, P-value: *p* < 0.001; Nagelkerke R^2^: 0.178.

### Reasons for not willing to take gastric cancer screening

**Figure 3:**
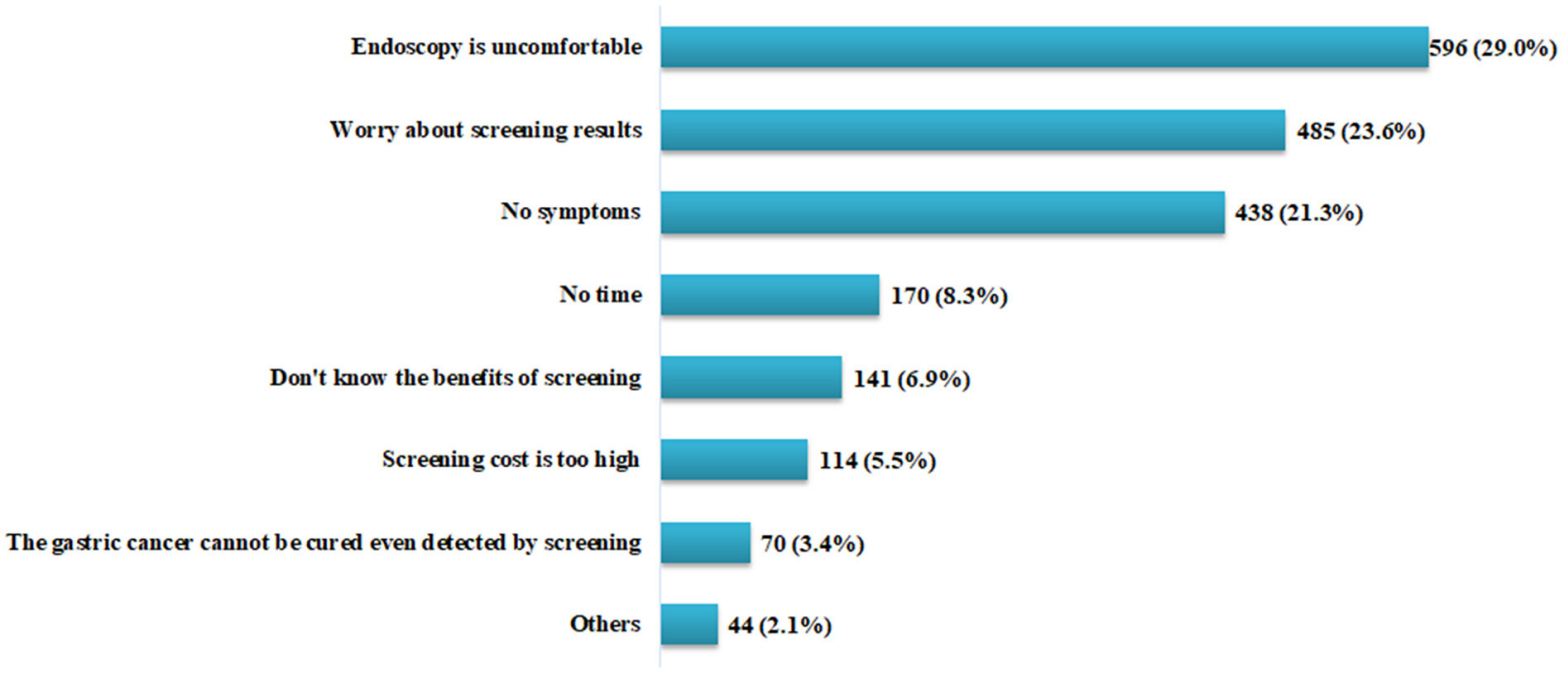
Reasons for not willing to have gastric cancer screening in the next 5 years (*N* = 2,058).

The domain reasons for not willing to take screening in the next 5 years are shown in Figure 3. Among respondents who reported probably yes/certainly no/probably no (*n* = 2,058), the three most common reasons, in descending order, were “endoscopy is uncomfortable” (29.6%), “worried about screening results” (23.6%), and “no symptoms” (21.3%). Other reasons included “no time” (8.3%), “don't know the benefits of screening” (6.9%), “screening cost is too high” (5.5%), and “believe that gastric cancer cannot be cured even detected by screening” (3.4%).

### Preferences of screening method

**Figure 4:**
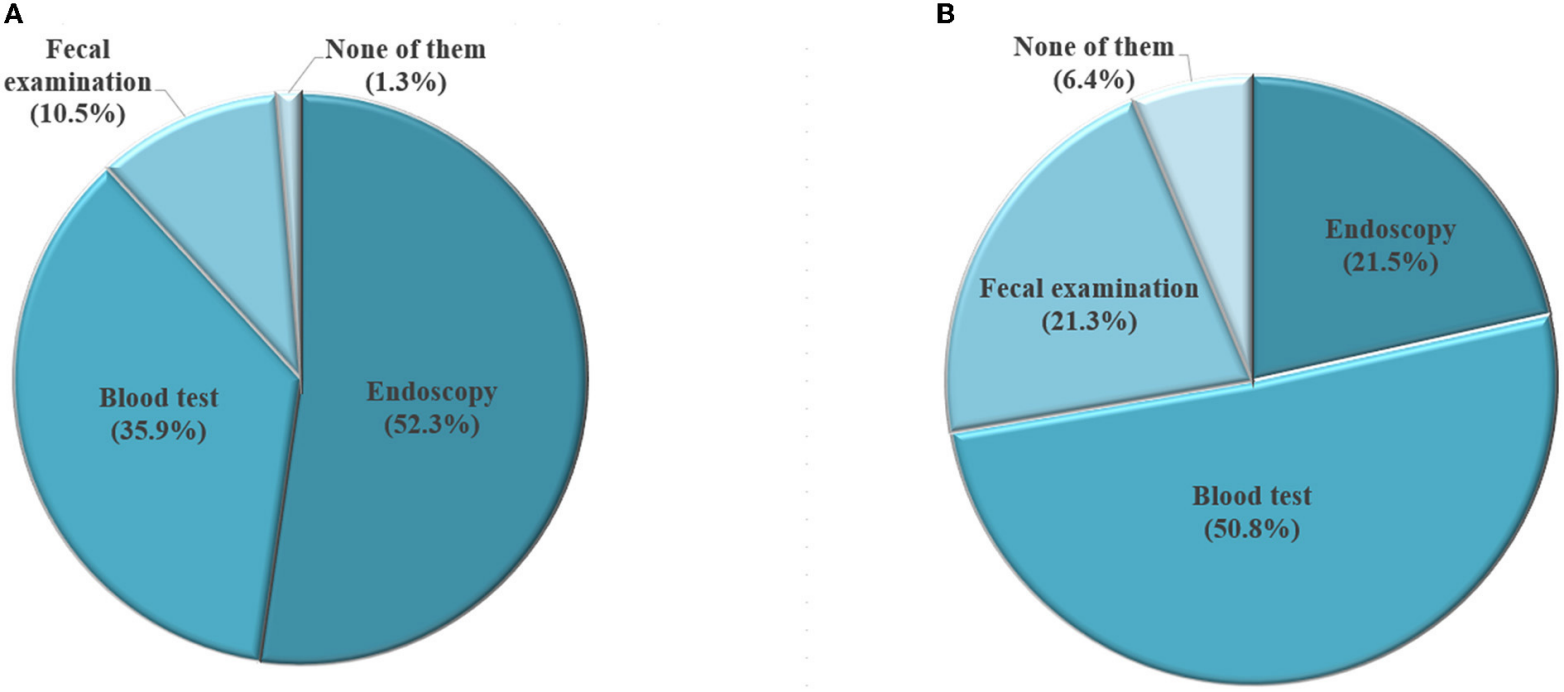
Selection of screening method among participants who have had gastric cancer screening (**A**, *N* = 1,086) and those who haven't had gastric cancer screening (**B**, *N* = 1,461).

Figure 4 presents respondents' preferences of screening method, grouping by if they ever took GC screening. For those who had ever taken GC screening, the most preferred screening method is endoscopy (52.3%), followed by blood test (35.9%), and fecal examination (10.5%). In contrast, among respondents who never took GC screening, the most favorite screening method was blood test (50.8%), followed by endoscopy (21.5%), and fecal examination (21.3%).

## Discussion

To our knowledge, the current study is the first investigation aimed to explore the knowledge level, attitudes to GC screening in high-risk populations in China. In general, the study participants reported a moderate level of knowledge in GC risk factors and warning symptoms. Majority of participants intended to take GC screening in the next 5 years. Significant factors influencing intention to screen were income level, previous history of GC screening or chronic gastric diseases, perceived overall health, total knowledge score, and HBM components (perceived benefit, perceived severity, perceived barriers, cues to action). “Endoscopy is uncomfortable,” “worry about screening results,” and “no symptoms” were the domain reasons for not willing to take screening.

Adequate knowledge about risk factors and warning symptoms of GC play an important role in cancer screening and early diagnosis. Poor knowledge about GC has been considered a barrier of GC screening (35). Result of our study also found that participants with high score of knowledge had a 50% increased intention to take GC screening. In 2015, China government implemented a Nationwide Three-Year Cancer Prevention Plan (2015–2017), announcing an ambitious goal to have the public awareness rate of essential cancer knowledge reach 60% (36). Our current study population in Southeastern China has shown a moderate level of knowledge. However, recent studies from other regions of China, including Central and Northeastern China, reported that people still have poor knowledge about GC (21, 37). More importantly, knowledge level varied among different types of risk factors. Specifically, participants were more familiar with life-style related risk factors, such as irregular diet habits, alcohol drinking, consumption of pickled/smoked foods, hot/spicy food, which is in line with a previous study (21). However, some imperative risk factors, such as male gender, family history of GC among first degree relatives were relatively rarely known. Finding from other previous study also identified these were the two least known risk factors (21). It seems that people tend to be more sensitive to those modifiable risk factors, but easily neglect unmodifiable factors such as age and heredity. Future health education program may need to particularly address high-risk populations under exposure to inherent risk factors. On the other hand, the need to improve knowledge about warning symptoms of GC is also clearly shown in the results of this study. In particular, findings indicate that a considerable proportion of surveyed participants lack knowledge of important symptoms such as early satiety and iron-deficiency anemia (IDA). IDA of gastrointestinal cancer origin is particularly common and longstanding due to bleeding. In the preoperative setting, a retrospective review by Jung et al. reported anemia in 43.6% (99/227) of GC patients. Of those, 24.2% (24/99) developed IDA (38). Recognition of warning signs was associated with anticipating faster help-seeking for potential symptoms of cancer (27). Knowing potential warning symptoms of GC may facilitate patients' treatment-seeking behavior.

Insight about demographic factors that facilitate or impede the intention to conduct GC screening may also be critical to promote health-behavior change. Multivariate analysis result of the current study implies that income level was the most robust factor associated with screening intention. High cost of endoscopy was also reported by surveyed participants as one of the major barriers toward screening. Similarly, Shin and Lee in a cross-sectional study reported that as the level of income increases, and the tendency to uptake screening also increases (OR = 1.36, 95% CI: 1.06–1.73) (29). Undoubtedly, affordability plays an important role in screening behavior. In China, endoscopy is conducted *via* opportunistic screening and individual own self is responsible for the related medical cost (39). Japan and Korea are the only two countries in the world that offer nationwide population-based GC screening (40). A Korean study shows that people were likely to intend to receive GC screening if it were offered free of charge or for a copayment (24). Our study also found participants were more likely to take GC screening if it is free of charge. Indeed, the screening rate in Korea has increased from 40.0% in 2005 to 74.8% in 2015 after the introduction of the National Cancer Screening Program which offer free or co-payment screening (41). Establishment of a population-based screening program to guarantee free access to endoscopy, particularly for high-risk populations, would be extremely critical for China and other high-risk regions to increase the early diagnosis rate of GC and consequently reduce the mortality rate.

Analysis results of HBM indicate that the following five components were significantly associated with screening intention, including perceived susceptibility (risk of getting GC is high), severity (afraid of getting GC), perceived benefit (GC screening is effective in saving life), perceived barriers (is uncomfortable), and cues-to-action (only take screening when it is free of charge). The finding of HBM could be utilized as a theoretical fundamental to design future health promotion program. In particular, discomfort related to endoscopy has been regarded as the most important reason for not being willing to take screening. Meanwhile, the majority of respondents without previous experience with endoscopic screening prioritized blood test for their future screening plan. These results implied that many people fear physical discomfort from the invasive endoscopy procedure. Although China government launched endoscopic screening program since 2005 in more than 110 high-risk areas throughout the country, the compliance rate (33.5%) was found to be low (20). To reduce the public's fear of endoscopy, recognition of its effectiveness for early detection of GC should be emphasized, and more efforts should be addressed to minimize the discomfort associated with the screening procedure. Alternative screening methods other than endoscopy could also be developed and implemented in order to improve the public's willingness to be screened. Furthermore, as *Helicobacter pylori* (a group I carcinogen) has been confirmed to have an important role in gastric carcinogenesis (42), people over 40 years old can be further stratified by *Helicobacter pylori* infection in order to find the most target population for endoscopic screening.

## Limitations

This study has several limitations that should be considered. The first pertains to the use of convenience sampling, in which the selection bias could not be eliminated, and its cross-sectional nature. It cannot, therefore, be used to infer causality. Second, data were collected from participants' self-reports; thus, these may be subjected to socially desirable responses. Third, it should be noted that the intention to take screening does not necessarily result in actual receipt of screening; therefore, results should be interpreted with caution. Fourth, the assessment of knowledge was done prior to screening intention, thus may potentially influence participants' responses to screening intentions. A final limitation of this study is that the study population was recruited from five major cities in Fujian province, which may limit generalizability. Despite these limitations, the study data contribute tremendously to the understanding of the influencing factors of GC screening intention in high-risk populations in China.

## Conclusions

The present study showed high intention to be screened for GC among high-risk populations aged 40 years and above in China, which is of great importance for a country with low GC screening coverage but high GC burden. Our results imply that economic factor might be the most robust indicator driving respondent's screening intention. To some degree, previous history of gastric diseases and GC screening, perceived overall health status, knowledge level related to GC risk factors and symptoms, and HBM components all contribute to decisions related to future screening intention. Population-based screening program is urgently needed to provide free access to screening, particularly for those high-risk populations. Additionally, continuous education campaigns are needed to improve knowledge of GC risk factors and symptoms in China and to promote the benefits of early cancer diagnosis by screening. Finally, more alternative screening methods other than endoscopy could also be encouraged to improve the general public's willingness to be screened.

## Data availability statement

The datasets used and/or analyzed during the current study are available from the corresponding author on reasonable request.

## Ethics statement

The studies involving human participants were reviewed and approved by Medical Ethics Committee at the Fujian Medical University. The patients/participants provided their written informed consent to participate in this study.

## Author contributions

ZHua: conceptualization, data curation, formal analysis, investigation, methodology, resources, software, validation, visualization, writing—original draft, and writing—review and editing. WL: data curation, investigation, and writing—review and editing. RM: writing—original draft and writing—review and editing. ZHu and LW: conceptualization, supervision, writing—original draft, and writing—review and editing. YL: conceptualization, data curation, formal analysis, investigation, methodology, resources, software, supervision, validation, visualization, funding acquisition, writing—original draft, and writing—review and editing. All authors contributed to the article and approved the submitted version.

## Funding

This study was supported by the National Natural Science Foundation of China (No.72004025). The funder had no role in study design, data collection and analysis, decision to publish, or preparation of the manuscript.

## Conflict of interest

The authors declare that the research was conducted in the absence of any commercial or financial relationships that could be construed as a potential conflict of interest.

## Publisher's note

All claims expressed in this article are solely those of the authors and do not necessarily represent those of their affiliated organizations, or those of the publisher, the editors and the reviewers. Any product that may be evaluated in this article, or claim that may be made by its manufacturer, is not guaranteed or endorsed by the publisher.
