# Supplementary material for: High-risk population's knowledge of risk factors and warning symptoms and their intention toward gastric cancer screening in Southeastern China
Source: Front Public Health. 2022 Aug 11;10:974923. doi: 10.3389/fpubh.2022.974923 (PMC9403326; doi:10.3389/fpubh.2022.974923)
Supplement: Supplementary file 1 [file Data_Sheet_1.PDF]

福建省城乡居民对胃癌认知水平的调查问卷【终版】

尊敬的先生/女士：

我们是福建医科大学公共卫生学院研究人员，想了解≥40 岁福建省福州市、泉州市、厦门市、漳州市、莆田市的城乡居民对于胃癌的相关危险因素、预警症状以及胃癌筛查的认知程度，以期在了解现状的基础上，指导之后的胃癌相关宣传教育、预防筛查、早诊断等，以期提高人群健康水平。您的参与，将为改善我国防癌相关卫生政策而做出重要的贡献，感谢您的参与！

请注意：此次调查为匿名问卷，我们将严格保存数据，采集的数据只限于课题相关人员进行科学研究。回答并提交此问卷，意味着您同意参加此次调查，并提交知情同意。

****请注意：本问卷填写对象为≥40 岁以上民众****

（一）一般情况

1.

年龄：___岁

身高：___厘米（cm）

体重：___公斤（kg） [填空题] *

2.民族 [单选题] *

○汉族

○其他 _________________

3.性别 [单选题] *

○男

○女

4.您目前的婚姻状况 [单选题] *

○未婚

○已婚

○离异或分居

○丧偶

5.您的受教育水平 [单选题] *

○小学及以下

○初中

○高中或中专

○大学及以上

6.职业 [单选题] *

○工人

○农民

○个体工商户

○专业技术人员(医护人员、教师、律师、记者等)

○机关、企事业单位管理者(干部)

○办事人员(普通公务员、公司职员等)

○服务业人员

○家庭主妇

○退休

○无业

○其他 (请填写) _________________

7.平均每月收入(元) [单选题] *

○<2000

○2000-5000

○≥5000

8.现工作、生活地点 [单选题] *

○城市

○近郊/县城

○农村

9.现工作、生活城市 [单选题] *

○福州

○莆田

○厦门

○漳州

○泉州

10.您是否有至亲(如父母、兄弟、姐妹等一级亲属)被诊断出胃癌？ [单选题] *

○是

○否

○不清楚

11.您是否有朋友、邻居、同事被诊断出胃癌？ [单选题] *

○是

○否

○不清楚

12.您抽烟吗？ [单选题] *

○是

○否

○曾经抽过，目前已戒烟 (至少半年或以上)

13.您是否有或曾经有饮酒习惯？(曾经在 6 个月内每周至少喝一次酒) [单选题] *

○是，现在仍喝酒

○以前喝酒，现在已戒

○偶尔喝酒，一周少于一次

○偶尔喝酒，一月少于一次

○不喝酒，一年少于一次

14.您是否有或曾经有饮茶习惯？(曾经在 6 个月内至少每天一杯) [单选题] *

○是，现在仍喝茶

○以前喝茶，现在已戒

○偶尔喝茶，一周少于一次

○不喝茶，一月少于一次

15.您是否有胃部相关疾病(比如慢性胃炎、胃溃疡等)？ [单选题] *

○是

○否

○不了解

16.您认为自己的健康状况是？ [单选题] *

○非常好

○很好

○挺好

○较差

○很差

17.您是否有医疗保险(省、市医保或者商业医疗保险) [单选题] *

○是

○否

○不了解

（二）对胃癌的认知和判断

18.您是否认为以下因素能增加患胃癌的风险？[矩阵单选题] *

	是	否	不了解
年龄在 40 岁以上	○	○	○
男性	○	○	○
幽门螺杆菌感染	○	○	○
胃溃疡	○	○	○
萎缩性胃炎	○	○	○
一级亲属（父母、子女、兄弟姐妹）有胃癌疾病史	○	○	○
高盐饮食	○	○	○
经常吃腌制食物	○	○	○
经常吃熏	○	○	○

制食物			
不规律饮食	○	○	○
经常吃剩饭菜	○	○	○
吸烟	○	○	○
饮酒	○	○	○
工作、生活压力大	○	○	○
经常吃宵夜	○	○	○
很少运动	○	○	○
很少吃蔬菜水果	○	○	○
较短或较长的睡眠时间	○	○	○
肥胖	○	○	○
有消化道系统疾病史	○	○	○
做过胃部手术	○	○	○

喜食辣食	○	○	○
喜食烫食	○	○	○
进食速度快	○	○	○

19.您认为此症状是否为胃癌的预警症状（提示可能发生胃癌）?[矩阵单选题] *

	是	否	不了解
消化道出血，如黑便、呕血等	○	○	○
持续恶心、呕吐	○	○	○
不明原因的体重骤减/消瘦	○	○	○
不明原因的疲倦、乏力	○	○	○
上腹部不适	○	○	○
上腹部肿块	○	○	○
上腹痛	○	○	○

食欲减退	○	○	○
吞咽困难或吞咽痛	○	○	○
早饱感（吃少于正常进食量就饱了）	○	○	○
反酸、呃逆	○	○	○
慢性胃炎久治不愈	○	○	○
缺铁性贫血	○	○	○
胃穿孔	○	○	○

（三）疾病诊疗习惯

20.您是否曾出现过持续性的恶心、呕吐、反酸、呃逆？ [单选题] *

○是

○否 (请跳至第 22 题)

21.如果您曾发生恶心、呕吐、反酸、呃逆，您大概是什么时间去就医？ [单选题] *

○三天内

○一周内

○两周内

○一个月内

○没有就医

22.您是否曾出现过持续性的食欲减退、乏力？ [单选题] *

○是

○否 (请跳至第 24 题)

23.如果您曾发生持续性的食欲减退、乏力，您大概是什么时间去就医？ [单选题] *

○三天内

○一周内

○两周内

○一个月内

○没有就医

24.您是否曾出现过黑便、便血？ [单选题] *

○是

○否 (请跳至第 26 题)

25.如果您曾发生过黑便、便血，您大概是什么时间去就医？ [单选题] *

○三天内

○一周内

○两周内

○一个月内

○没有就医

26.您是否出现过不明原因的体重突然骤减/消瘦？ [单选题] *

○是

○否 (请跳至第 28 题)

27.如果您曾发生过体重突然骤减/消瘦，您大概是什么时间去就医？ [单选题] *

○三天内

○一周内

○两周内

○一个月内

○没有就医

28.您是否曾经患有上述任一症状？ [单选题] *

○是

○否 (请跳至第 30 题)

29.当您患有以上症状时，您去寻求医生诊治的难易度？ [单选题] *

○非常困难

○比较困难

○比较容易

○非常容易

30.以下哪些会是您推迟去看医生的原因？ [多选题] *

□看病太贵

□医生很难预约

□我担心会浪费医生的时间

□我担心医生会发现我什么疾病

□我太忙了，没有时间去看医生

□其他 _________________*

31.当医生建议您去做胃镜、胃癌标识物检测等进一步胃癌筛查检测时，您是否对寻求医生诊治有困难？ [单选题] *

○是

○否

○不了解/无相关经历

（五）胃癌筛查知识及态度

32.您认为胃癌是能被预防的吗？ [单选题] *

○是

○否

○不了解

33.您认为早期胃癌能治好吗? [单选题] *

○是

○否

○不了解

34. 您是否做过胃癌筛查（胃镜筛查）？ [单选题] *

○是

○否

○不了解

35. 您认为筛查对于检出早期胃癌是否有用？ [单选题] *

○是

○否

○不了解

36. 下列哪种筛查方式您更能接受？ [单选题] *

○胃镜

○血液

○粪便检查

○没有一项

37. 在接下来的 5 年中，您打算去做胃癌筛查（胃镜筛查）吗？ [单选题] *

○绝对不会

○可能不会

○可能会

○绝对会 (请跳至第 39 题)

38. 不参加胃癌筛查的主要原因？ [单选题] *

○不知道检查有什么好处

○做胃镜难受

○怕查出病，有心理负担

○没有症状，不需要检查

○查出也治不好，不如不查花钱

○没有时间

○价格太贵

○其他 _________________ *

39. 您目前生活工作所在地？ [单选题] *

○城市

○近郊/县城

○农村

（六）健康信念

40. 胃癌的易感性[矩阵单选题] *

	强烈同意	同意	不同意	强烈不同意	不了解
一般来说，人的一生中患胃癌的几率很大	○	○	○	○	○
我有可能会患胃癌	○	○	○	○	○
一般来说，人的一生中感染幽门螺旋杆菌的风险很高	○	○	○	○	○

41. 胃癌严重程度的认知[矩阵单选题] *

	强烈同意	同意	不同意	强烈不同意	不了解
胃癌对人的伤害是很严重的	○	○	○	○	○
胃癌患者的死亡率很高	○	○	○	○	○
我害怕患上胃癌	○	○	○	○	○

42. 胃癌筛查的益处[矩阵单选题] *

	强烈同意	同意	不同意	强烈不同意	不了解
胃癌筛查是很有好处的，因为可以帮助胃癌的早诊断、早治疗	○	○	○	○	○
胃癌筛查会降低我死于胃癌的几率，或提高生存率	○	○	○	○	○

43. 胃癌筛查的障碍[矩阵单选题] *

	强烈同意	同意	不同意	强烈不同意	不了解
我担心胃癌筛查会发现什么	○	○	○	○	○
只有在有症状时，才需要进行胃癌筛	○	○	○	○	○

查					
胃镜检查很难受	○	○	○	○	○
我担心胃镜检查费用太高	○	○	○	○	○
我担心预约不到胃镜检查，或者要等很久	○	○	○	○	○

44. 胃癌筛查的前提[矩阵单选题] *

	强烈同意	同意	不同意	强烈不同意	不了解
胃癌筛查是免费时，我才会去做	○	○	○	○	○
胃癌筛查可以纳入医保报销时，我才会去做	○	○	○	○	○
医生建议我去胃癌	○	○	○	○	○

筛查时，我才会去做					

45. 您的出生年月是：__________ 年 ___ 月 [填空题] *

问卷到此结束，衷心感谢您的支持！
