# Supplementary material for: High-risk population's knowledge of risk factors and warning symptoms and their intention toward gastric cancer screening in Southeastern China
Source: Front Public Health. 2022 Aug 11;10:974923. doi: 10.3389/fpubh.2022.974923 (PMC9403326; doi:10.3389/fpubh.2022.974923)
Supplement: Supplementary file 2 [file Table_1.DOCX]

**Supplemental Table 1**. Comparison of demographic characteristics of the study population and the general adults population in Fujian province, 2021.

| **Age groups** |  | **% Study population**  **N=2547** | | | **% Fujian population N=19282907**† | | |
| --- | --- | --- | --- | --- | --- | --- | --- |
|  | N | Total^‡^ | Male^¶^ | Female^¶^ | Total^‡^ | Male^¶^ | Female^¶^ |
| 40-44 years | 912 | 35.8% | 61.1% | 38.9% | 15.6% | 51.3% | 48.7% |
| 45-49 years | 916 | 36.0% | 61.6% | 38.4% | 18.5% | 51.1% | 48.9% |
| 50-54 years | 341 | 13.4% | 58.1% | 41.9% | 17.4% | 50.9% | 49.1 |
| 55-59 years | 204 | 8.0% | 58.3% | 41.7% | 14.0% | 50.4% | 49.6% |
| 60-64 years | 67 | 2.6% | 47.8% | 52.2% | 10.5% | 50.4% | 49.6% |
| 65-69 years | 57 | 2.2% | 47.7% | 52.6% | 9.4% | 48.9% | 51.1% |
| 70-74 years | 24 | 0.9% | 45.8% | 54.2% | 5.9% | 49.8% | 50.2% |
| 75-79 years | 11 | 0.4% | 27.3% | 72.7% | 3.7% | 48.6% | 51.4% |
| ≥80 years | 15 | 0.6% | 73.3% | 26.7% | 4.9% | 42.1% | 57.9% |

† Total number of adults aged 40years and older according to Fujian census in 2021;

‡ Percentage of each age group of total population;

¶ Percentage withing the age group.
